# Supplementary material for: COOBoostR: An Extreme Gradient Boosting-Based Tool for Robust Tissue or Cell-of-Origin Prediction of Tumors
Source: Life (Basel). 2022 Dec 27;13(1):71. doi: 10.3390/life13010071 (PMC9865194; doi:10.3390/life13010071)
Supplement: Supplementary file 1 [file life-13-00071-s001.zip › life-2081510-supplementary.pdf]

Supplemental Figure S1

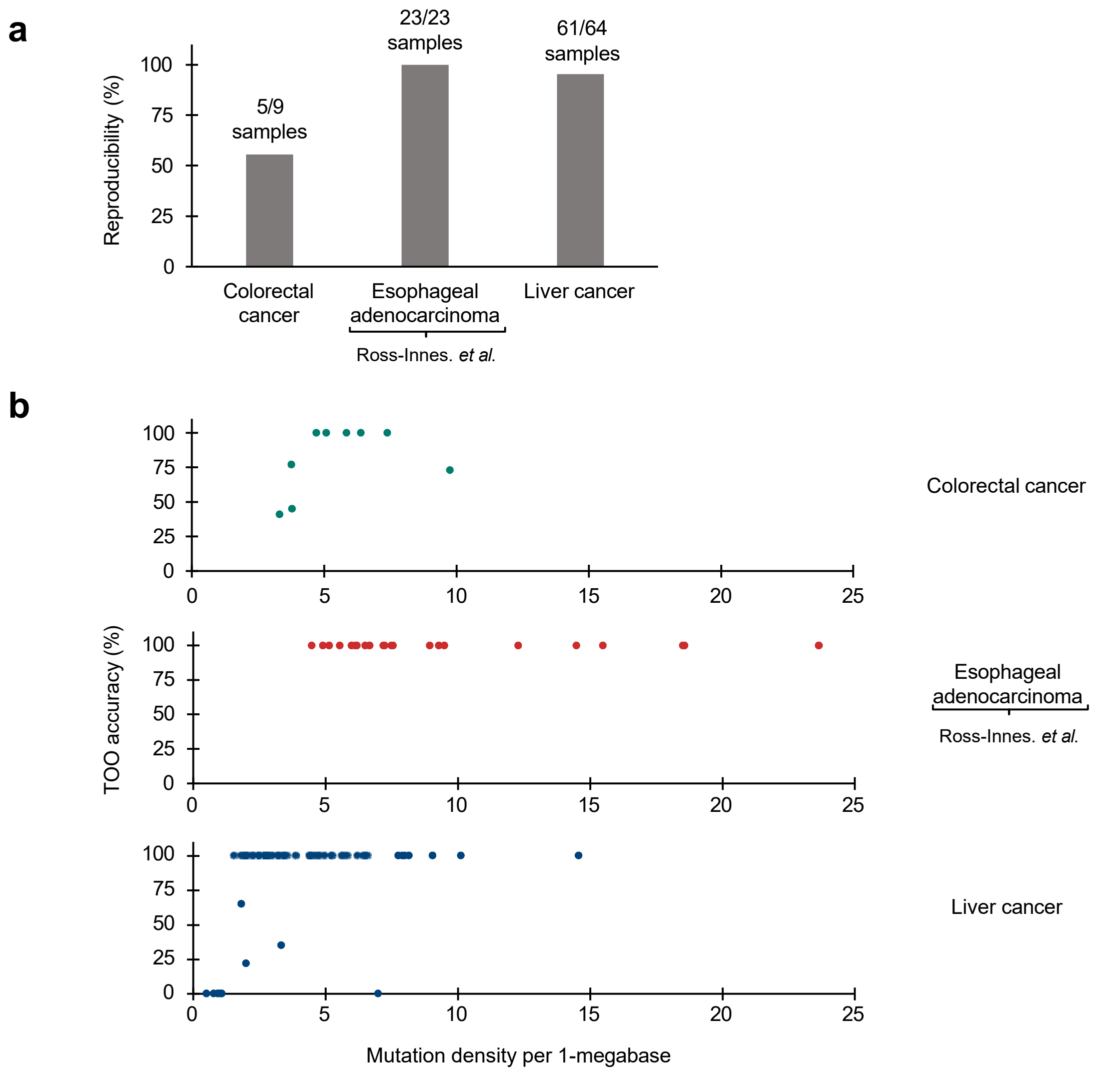

Supplemental Figure S1. TOO accuracy comparisons between COOBoostR and Random forest-based algorithm. (**a**) Reproducibility of COOBoostR. The number of samples showing either 0% or 100% accuracy is represented above the bar graph. (**b**) TOO prediction accuracy for colorectal cancer, esophageal adenocarcinoma, and liver cancer at an individual sample level using 100 repeated COOBoostR algorithm. Samples are aligned in order of mutation density magnitude per 1-megabase window.

Supplemental Figure S2

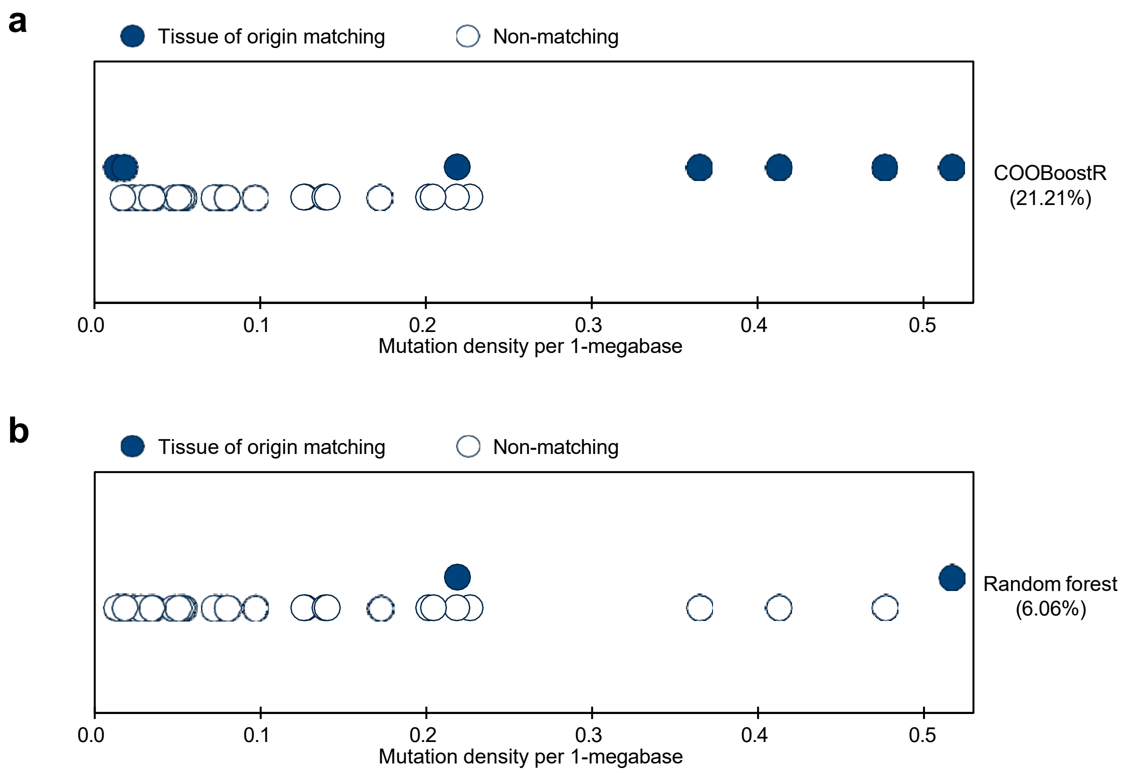

Supplemental Figure S2. TOO prediction accuracy for hepatoblastoma at an individual sample level using (**a**) COOBoostR and (**b**) Random forest-based algorithm. Samples matching predicted TOO are marked with solid circles, and samples that did not match are marked with empty circles. Dots were jittered to dissect out the blue and white dots. Samples are aligned in order of mutation density magnitude per 1-megabase window.

Supplemental Figure S3

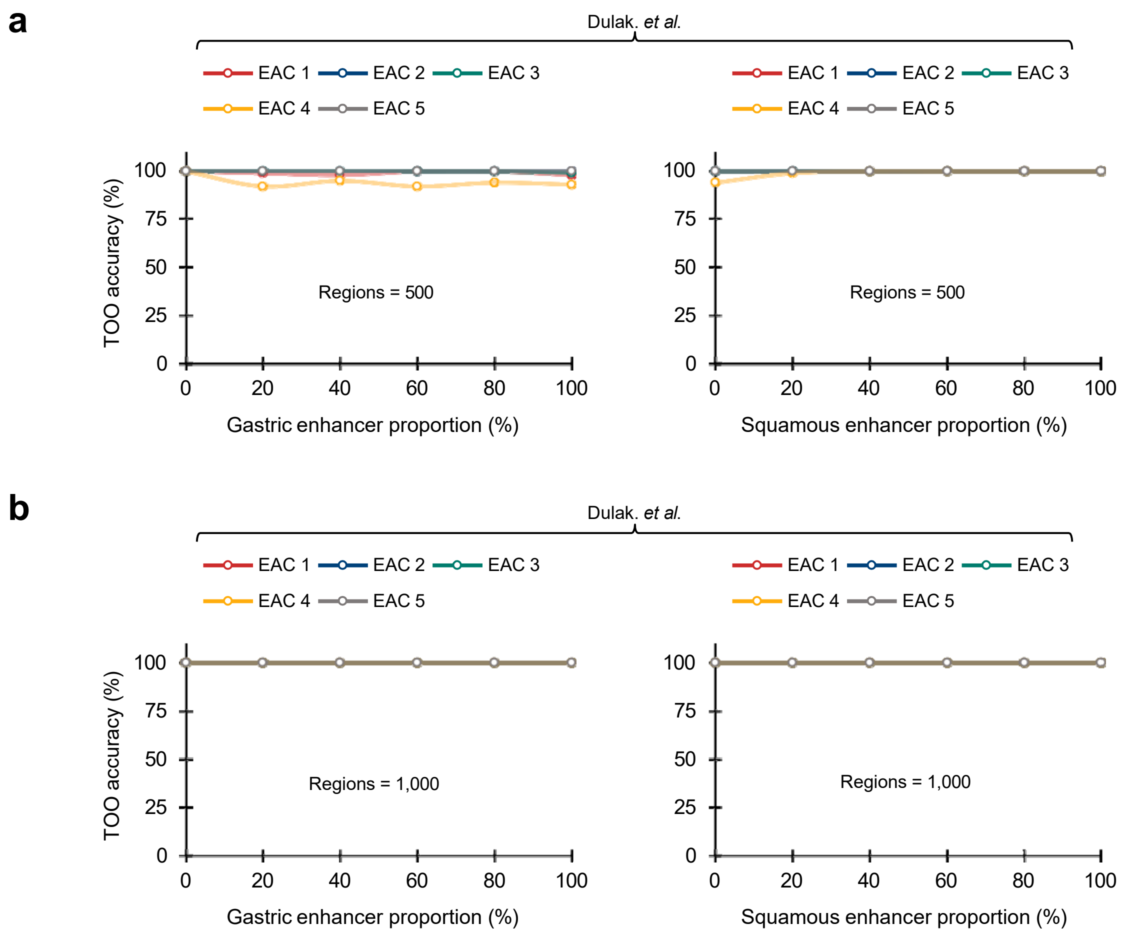

Supplemental Figure S3. Region selection analysis with respect to the portion of gastric / squamous specific enhancer containing regions for EACs at individual sample level. Enhancer inclusion ratio was varying from 0 to 100%, making up to (**a**) 500 and (**b**) 1,000 regions.
